# Intrahepatic neutrophil accumulation and extracellular trap formation are associated with posthepatectomy liver failure

**DOI:** 10.1097/HC9.0000000000000348

**Published:** 2023-12-15

**Authors:** Laura Brunnthaler, David Pereyra, Miriam Brenner, Jonas Santol, Lukas Herrmann, Waltraud C. Schrottmaier, Anita Pirabe, Anna Schmuckenschlager, Sarang Kim, Anna Emilia Kern, Felix Xaver Huber, Lisa Emilie Michels, Christine Brostjan, Manuel Salzmann, Philipp Hohensinner, Renate Kain, Thomas Gruenberger, Patrick Starlinger, Alice Assinger

**Affiliations:** 1 Department of Vascular Biology and Thrombosis Research, Centre of Physiology and Pharmacology, Medical University of Vienna, Vienna, Austria; 2 Department of General Surgery, Division of Visceral Surgery, Medical University of Vienna, General Hospital, Vienna, Austria; 3 Department of Surgery, HPB Center, Viennese Health Network, Clinic Favoriten and Sigmund Freud Private University, Vienna, Austria; 4 Department of Medicine II, Division of Cardiology, Medical University of Vienna, General Hospital, Vienna, Austria; 5 Center for Biomedical Research, Division of Biomedical Research, Medical University of Vienna, Vienna, Austria; 6 Department of Pathology, Medical University of Vienna, General Hospital, Vienna, Austria; 7 Department of Surgery, Division of Hepatobiliary and Pancreatic Surgery, Mayo Clinic, Rochester, Minnesota, USA

## Abstract

**Background:**

Posthepatectomy liver failure (PHLF) represents a life-threatening complication with limited therapeutic options. Neutrophils play a critical and dynamic role during regeneratory processes, but their role in human liver regeneration is incompletely understood, especially as underlying liver disease, detectable in the majority of patients, critically affects hepatic regeneration. Here we explored intrahepatic neutrophil accumulation and neutrophil extracellular traps (NETs) in patients with PHLF and validated the functional relevance of NETs in a murine partial hepatectomy (PHx) model.

**Methods:**

We investigated the influx of neutrophils, macrophages, eosinophils, and mast cells and the presence of their respective extracellular traps in liver biopsies of 35 patients undergoing hepatectomy (10 patients with PHLF) before and after the initiation of liver regeneration by fluorescence microscopy. In addition, NET formation and neutrophil activation were confirmed by plasma analysis of 99 patients (24 patients with PHLF) before and up to 5 days after surgery. Furthermore, we inhibited NETs via DNase I in a murine PHx model of mice with metabolically induced liver disease.

**Results:**

We detected rapid intrahepatic neutrophil accumulation, elevated levels of myeloperoxidase release, and NET formation in regenerating human livers, with a significantly higher increase of infiltrating neutrophils and NETs in patients with PHLF. Circulating markers of neutrophil activation, including elastase, myeloperoxidase, and citrullinated histone H3, correlated with markers of liver injury. In a murine PHx model, we showed that the inhibition of NET accelerated hepatocyte proliferation and liver regeneration.

**Conclusions:**

Patients with PHLF showed accelerated intrahepatic neutrophil infiltration and NET formation, which were associated with liver damage. Further, we identified postsurgical myeloperoxidase levels as predictive markers for adverse outcomes and observed that blocking NETs in a murine PHx model accelerated tissue regeneration.

## Graphical abstract

**Figure:**
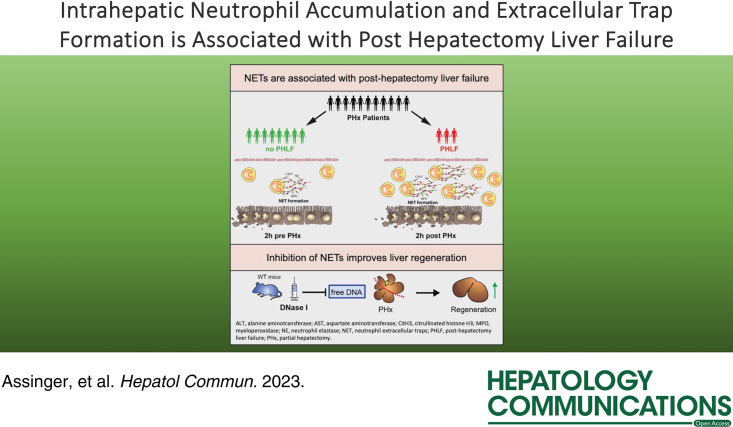

## INTRODUCTION

Due to environmental and lifestyle factors as well as increased life expectancies, a dramatic increase in patients experiencing persistent liver injuries, with hepatic scarring, cirrhosis, and ultimately liver cancer, occurred during the past decades, making liver cancer the most rapidly increasing tumor entity.^1^ The liver bears the unique capacity to regenerate after injuries.^2^ Therefore, liver resection often represents an attractive curative treatment option in patients with primary liver cancer or liver metastasis. However, underlying liver disease complicates liver regeneration processes leading to postsurgical liver dysfunction and consequent liver failure remains a frequent sequel after hepatic resection, causing morbidity and mortality.^3^ Curative treatment options of posthepatectomy liver dysfunction are limited and the discovery of new therapeutic interventions is crucial to improving postsurgical organ function.^4^

Upon injury, hepatocytes release various damage-associated molecular patterns (DAMPs) which together with rheological changes foster the recruitment of neutrophils.^5^ Infiltrating neutrophils aid pathogen removal and repair of the liver tissue after hepatic resection. Hemodynamic changes after liver resection, which increase the presence of cytokines, growth factors, platelets, and leukocytes in the liver, can trigger neutrophil extracellular trap (NET) formation,^6^ a neutrophil-related programmed cell death, characterized by web-like structures with antimicrobial proteins such as myeloperoxidase (MPO) and neutrophil elastase (NE). NETs entrap circulating bacteria,^7^ but are also associated with host damage and microthrombosis.^8^ Moreover, the liver serves as a possible filter for free-floating NETs as it retains NETs in the liver vasculature system due to high von Willebrand factor binding to histones.^9^

While neutrophils have clear beneficial roles in liver homeostasis, excessive neutrophil activation and NET formation have been connected to the pathophysiology of various liver diseases.^10^ There is strong evidence for a detrimental role in NASH, portal hypertension, alcohol-associated liver disease, ischemia/reperfusion injury, and PVT.^11–14^ NETs may promote tumorigenesis, angiogenesis, and thrombus formation in various cancer types including HCC^15^ and contribute to the progression of acute liver failure.^16^

While inflammation is essential for the initiation of liver regeneration, the role of neutrophil activation and NET formation in posthepatectomy liver regeneration is to date unknown.^10^ Therefore, we investigated neutrophil accumulation and NET formation in liver biopsies from patients with and without posthepatectomy liver failure (PHLF) before and after surgery and monitored circulating markers of neutrophil activation and NETs up to 5 days after surgery. We found excessive neutrophil accumulation and NET formation to be associated with adverse outcomes. Our data provide evidence that NETs are potentially associated with PHLF and that postsurgical MPO levels serve as predictive markers for adverse outcomes. Using a murine partial hepatectomy (PHx) model of metabolically challenged mice with sterile liver inflammation to mimic the human situation, we could further demonstrate that the inhibition of NETs via DNase I treatment could restore the hepatic regeneratory capacity.

## METHODS

### Patient cohort

Tissue and plasma samples of 99 patients undergoing PHx between March 2011 and May 2020 at 3 Austrian institutions (General Hospital of Vienna, Clinic Landstrasse, and Clinic Favoriten) were collected out of a prospectively maintained biobank. Patients had to have compensated liver function accessed by ICG clearance or LIMAX; additionally, all patients had to have a Child-Pugh Score A to undergo surgery. Exclusion criteria included pregnancy, age below 18 years, and intraoperative abortion of surgery due to disease progression. The study was conducted in adherence to the Declaration of Helsinki and was approved by the responsible Institutional Ethics Committee. Ahead of participation, informed consent was obtained from all patients (EK 16-253-0117, EK 14-122-0714). Patients were followed prospectively over a postoperative time period of 5 days. Blood samples were taken and assessed 1 day before surgery (PreOP) as well as 1 day (POD1) and 5 days after surgery (POD5). Routine laboratory parameters as well as sex, age, tumor type, routine preoperative parameters, morbidity, and classification of PHLF via ISGLS (International Study Group of Liver Surgery) grading^17^ were assessed.

In a subset of patients undergoing PHx, intraoperative tissue samples were obtained before and 2 hours after ligation of the portal branch indicating the starting point for the induction of liver regeneration. For further analyses, all 10 patients with PHLF were included and 25 matched noPHLF were selected according to sex, age, and underlying liver comorbidities.

### Plasma preparation

Plasma preparation was performed as described.^18^ Whole-blood samples were collected into prechilled CTAD (citrate, theophylline, adenosine, and dipyridamole) tubes and immediately placed onto ice, and further processed within 30 minutes. Following an initial centrifugation at 1000*g* at 4°C for 10 minutes, supernatants were centrifuged for 10,000*g* at 4°C for 10 minutes to remove the remaining platelets. Plasma samples were stored in aliquots at −80°C until analysis.

### Definition and classification of postoperative liver dysfunction and complications

The definition and classification of PHLF were assessed according to the criteria of the ISGLS.^17^ Notably, blood from patients who were discharged earlier or reached normal serum bilirubin or prothrombin time values before POD5 was not further collected and the patient was classified as displaying “normal functional liver regeneration.”

### Plasma analysis for MPO, NE, and citrullinated histone H3

MPO levels were quantified using the commercially available Human Myeloperoxidase Quantikine ELISA Kit (R&D Systems) and NE levels were assessed by the commercially available Human PMN (Neutrophil) Elastase ELISA Kit (Invitrogen) according to the manufacturer’s instructions.

The quantification of citrullinated histone H3 (CitH3) levels in human plasma was based on the Cell Death Detection Kit (Roche), adjusted in accordance with a published protocol from Thålin et al.^19^

### Animal treatments

All animal experiments were approved by the Austrian Federal Ministry for Science, Research and Economy (BMBWF-V/3b/2023-0.188.679). Five-week-old wild-type C57BL/6J mice were put on a high-saturated fat/cholesterol diet (AIN-76 Western Diet, Test Diet), and water was supplemented with sucrose/fructose (42 g/L) for 10 weeks, referred to as fast food diet (FFD). Fifteen-week-old mice on FFD were treated intraperitoneally with either 100 µL PBS (n=7) or DNase I (120 µL i.v., Sigma 10104159001, 1.8 mg/mL, n=6) intravenously 30 minutes before the operation, followed by 2 injections the following day and one last injection was administered 2 hours before sacrificing the mice 48 hours after PHx. Liver-to-bodyweight ratio as well as liver recovery rate were calculated. Using FUJI DRI-CHEM NX500 (Lab Technologies), aspartate aminotransferase or alanine aminotransferase (ALT) measurements of murine plasma were carried out.

### Surgical procedure

PHx operation was performed according to the 2/3 PHx model, described by Mitchell and Willenbring.^20^ Mice were anesthetized with 2% isoflurane and injected with 10 µL/g tramadolhydrochlorid for analgesia. A midabdominal skin incision was done and surrounding ligaments and membranes were divided. First, the gall bladder was removed. Afterward, the left-lateral lobe was resected with ligation at its base, and the median lobe was resected with ligation at the level between the gall bladder and the suprahepatic inferior vena cava. Next, the abdomen was closed and the mice were placed on a warming pad until they recovered from anesthesia. Two hours after surgery, 10 µL/g tramadolhydrochlorid was injected for analgesic management followed by another injection every 12 hours for 2 days, according to the institutional guidelines. After surgery, mice were maintained with free access to water and food in temperature-controlled conditions and sacrificed after 48 hours.

### Immunofluorescence staining

In a subset of either 35 (NETs) or 25 [leukocytes, macrophage extracellular traps, mast cell extracellular traps, and eosinophil extracellular traps] patients, liver samples before and 2 hours after ligation were analyzed for extracellular trap formation by immunofluorescence microscopy. Paraffin-embedded liver biopsies were deparaffinized and permeabilized (0.1% Triton X-100) after heat-mediated antigen retrieval (citrate buffer pH 6.0). Mouse livers before and after PHx were cryo-embedded and permeabilized (0.1% Triton X-100). After blocking (10% fetal calf serum) primary antibodies were incubated overnight at 4°C and secondary antibodies were incubated for 2 hours at room temperature (Supplemental Table S1, http://links.lww.com/HC9/A700). Nuclei were counterstained using Hoechst 33342 and slides were mounted with ProlongGold Antifade mounting reagent. Microscope images were obtained via Widefield Fluorescence Nikon A1plus Ti Microscope and suitable scale bars were added in ImageJ Fiji. Data analysis of the obtained images was performed via CellProfiler 3.1.9.

### RNA extraction and real-time quantitative PCR analysis

Total RNA from harvested livers was isolated using TriFast reagent according to the manufacturer’s protocol (VWR). One microgram of RNA was reverse transcribed using the High Capacity cDNA Reverse Transcription Kit (Thermo Fisher Scientific) according to the manufacturer’s instructions. Quantitative real-time PCR was performed on a Bio-Rad CF X96 Real-Time System (Bio-Rad) using the GoTaq qPCR Mastermix (Promega). Genes of interest were normalized to hypoxanthine-guanine phosphoribosyltransferase (Hprt) expression as the reference gene. Expression profiles and associated statistical parameters were determined by the 2−ΔΔCT method. Specific oligonucleotide primers are shown in Supplemental Table S2, http://links.lww.com/HC9/A700.

### Statistical evaluation

Statistical analyses were carried out with either Graphpad Prism 8.0.2 (GraphPad Software Inc.) or IBM SPSS Statistics 20 (SPSS, Inc.). Gaussian distribution was analyzed via Anderson Darling (A2*), D’Agostino-Pearson omnibus (K2), Shapiro-Wilk (W), and Kolmogorov-Smirnov (distance) tests. If data followed Gaussian distribution, time point comparisons were analyzed via a paired two-tailed *t* test. If Gaussian distribution was not given, a two-tailed Wilcoxon matched-pairs signed-rank test was applied. For the comparison of the patient groups, PHLF and no PHLF, and their differences at time points, baseline and regeneration, a two-way ANOVA test with Tukey correction for multiple comparisons was applied. A CI of 95% was calculated in all used test systems. Further, a *p* value of <0.05 was considered as a significant difference between groups.

## RESULTS

### Patient characteristics

A total of 99 patients undergoing hepatic resection were included in this study, of which 24 developed PHLF, and analyzed for plasma parameters (Table 1). Out of these patients, 25 (PHLF=7) and 35 (PHLF=10) patients were used for immunofluorescence staining. Patient characteristics of plasma and staining cohorts are summarized in Tables 1 and 2. Patients with PHLF indicate no correlation with sex, preoperative parameters, and hepatic comorbidities including chemotherapy, steatosis, steatohepatitis, fibrosis, and hepatitis. Nonetheless, a significantly higher proportion of patients with PHLF underwent major resection and presented with metastatic colorectal cancer. Furthermore, they were associated with morbidity grades IV and V, and more total hospitalization days.

**TABLE 1:** Plasma cohort patient characteristics

Parameter	Cohort (n=99)	PHLF cohort (n=24)	No PHLF cohort (n=75)	Missing values (%)
Sex, n (%)				
Male	65 (65.7)	13 (54.2)	52 (69.3)	
Female	34 (34.3)	11 (45.8)	23 (30.7)	
Age (y)	62.5 (22.0–86.0)	62.4 (35.0–80.7)	62.6 (22.0–86.0)	
Hepatic resection, n (%)				
**Minor (<3 segments)**	26 (26.3)	2 (8.3)	24 (32.0)	
**Major (≥ 3 segments)**	73 (73.7)	22 (91.7)	51 (68.0)	
Tumor type, n (%)				
**mCRC**	34 (34.3)	4 (16.7)	30 (40.0)	
HCC	29 (29.3)	5 (20.8)	24 (32.0)	
**CCC**	21 (21.2)	10 (41.7)	11 (14.6)	
Pancreas related	8 (8.1)	3 (12.5)	5 (6.7)	
Others	7 (7.1)	2 (8.3)	5 (6.7)	
Hepatic comorbidities				
Neoadjuvant CTx, n (%)	34 (35.7)	5 (21.7)	19 (26.4)	4 (4.0)
Steatosis (%)	14.0 (0.0–100.0)	6.8 (0.0–40.0)	17 (0.0–100.0)	
Steatohepatitis, n (%)	25 (26.0)	4 (18.2)	21 (28.4)	3 (3.0)
Fibrosis, n (%)	55 (57.3)	11 (52.4)	44 (58.7)	3 (3.0)
CASH, n (%)	19 (19.6)	3 (13.6)	16 (21.3)	2 (2.0
Intraoperative RBCs transfusion, n (%)	10 (10.1)	4 (16.7	6 (8.0)	
Hepatitis, n (%)	8 (8.1)	1 (4.2)	7 (9.3)	
Antivirals, n (%)	3 (3.0)	0 (0.0)	3 (4.0)	
Preoperative parameters				
PDR (%)	20.9 (9.0–40.0)	21.3 (9.7–40.0)	20.8 (9.0–38.3)	24 (24.2)
Platelets (×10^3^/µL)	248.7 (86.0–487.0)	262.4 (172.0–345.0)	237 (86.0–487.0)	10 (10.1)
SB (mg/dL)	0.8 (0.2–6.6)	1.0 (0.2–6.6)	0.7 (0.2–6.6)	13 (13.1)
PT (%)	104.6 (40.0–150.0)	97.2 (40.0–150.0)	107.0 (74.0–150.0)	18 (18.2)
AP (U/L)	129.7 (46.0–707.0)	162.8 (51.0–707.0)	100.0 (46.0–314.0)	16 (16.2)
GGT (U/L)	147.2 (7.0–1576.0)	211.3 (18.0–1576)	127 (7.0–710.0)	11 (11.1)
AST (U/L)	45.4 (14.0–242.0)	53.9 (14.0–224.0)	44.0 (17.0–242.0)	28 (28.3)
ALT (U/L)	45.97 (8.0–372.0)	44.2 (12.0–129.0)	48.0 (8.0–372.0)	11 (11.1)
Albumin (g/L)	41.4 (32.5–47.6)	38.8 (32.5–47.3)	43.2 (34.4–47.6)	19 (19.2)
Morbidity, n (%)				
**No morbidity**	46 (46.5)	4 (16.7)	42 (56.0)	
Grade I	12 (12.1)	2 (8.3)	10 (13.3)	
**Grade II**	19 (19.2)	7 (29.2)	12 (16.0)	
Grade III	16 (16.2)	6 (25.0)	10 (13.3)	
**Grade IV**	2 (2.0)	2 (8.3)	0 (0.0)	
**Grade V**	4 (4.0)	3 (12.5)	1 (1.4)	
Postoperative stay				
ICU (d)	2.1 (0.0–15.0)	2.9 (0.0–15.0)	2 (0.0–15.0)	
**Total hospitalization (d)**	13.6 (4.0–75.0)	19.5 (5.0–56.0)	12.0 (4.0–75.0)	
PHLF ISGLS, n (%)				
**No PHLF**	75 (75.7)		75 (100.0)	
**Grade A**	6 (6.1)	6 (25.0)		
**Grade B**	7 (7.1)	7 (29.2)		
**Grade C**	11 (11.1)	11 (45.8)		

*Note*: Values in bold: PHLF vs. noPHLF: *p*>0.05.

Abbreviations: ALT, alanine aminotransferase; AP, alkaline phosphatase; AST, aspartate aminotransferase; CCC, cholangiocellular carcinoma; CASH, chemotherapy-induced acute steatohepatitis; CTx, chemotherapy; GGT, gamma-glutamyl transpeptidase; ICU, intensive care unit; ISGLS, International Study Group of Liver Surgery; mCRC, metastatic colorectal cancer; PDR, plasma disappearance rate; PHLF, posthepatectomy liver failure; PT, prothrombin time; RBCs, red blood cells; SB, serum bilirubin.

**TABLE 2:** Staining cohort patient characteristics

Parameter	Subcohort (n=35)	PHLF cohort (n=10)	No PHLF cohort (n=25)	Missing values, n (%)
Sex, n (%)				
Male	17 (48.6)	6 (60.0)	11 (44.0)	
Female	18 (51.4)	4 (40.0)	14 (56.0)	
Age (y)	58.7 (35.0–80.0)	59.6 (35.0–76.7)	58.4 (41.2–80.0)	
Hepatic resection, n(%)				
Minor (<3 segments)	0 (0.0)	0 (0.0)	0 (0.0)	
Major (≥3 segments)	35 (100.0)	10 (100.0	25 (100.0)	
Tumor type, n (%)				
mCRC	11 (31.4)	3 (30.0)	8 (32.0	
HCC	9 (25.7)	1 (10.0)	8 (32.0%)	
**CCC**	4 (11.4)	4 (40.0)	0 (0.0)	
Pancreas related	4 (11.4)	2 (20.0)	2 (8.0)	
Others	7 (20.0)	0 (0.0)	7 (28.0)	
Hepatic comorbidities				
Neoadjuvant CTx, n (%)	15 (42.9)	4 (40.0)	11 (44.0)	
Steatosis (%)	4.6 (0.0–25.0)	3.5 (0.0–10.0)	5.4 (0.0–25.0)	
Steatohepatitis, n (%)	13 (37.1)	2 (20.0)	11 (44.0)	
Fibrosis, n (%)	13 (40.6)	3 (42.9)	10 (40.0)	3 (8.6)
CASH, n (%)	10 (28.6)	4 (40.0)	6 (24.0)	
Intraoperative RBCs, n (%)	5 (14.3)	3 (30.0)	2 (8.0)	
Hepatitis, n (%)	1 (2.9)	0 (0.0)	1 (2.9)	
Antivirals, n (%)	0 (0.0)	0 (0.0)	0 (0.0)	
Preoperative parameters				
PDR (%)	22.1 (9.0–40.0)	24.0 (20.0–32-0)	21.8 (9.0–40.0)	10 (28.6)
Platelets (×10^3^/µL)	266.6 (117.0–487.0)	240.3 (172.0–333.0)	276.1 (117.0–487.0)	4 (11.5)
SB (mg/dL)	0.7 (0.2–2.5)	0.6 (0.2–1.2)	0.8 (0.2–2.5)	6 (17.1)
PT (%)	102.7 (40.0–133.0)	100.4 (73.0–123.0)	105.4 (40.0–133.0)	9 (25.7)
AP (U/L)	116.5 (48.0–707.0)	155.9 (65.0–142.0)	89.0 (48.0–707.0)	9 (25.7)
GGT (U/L)	135.1 (7.0–1576.0)	226.2 (27.0–1576.0)	87.2 (7.0–360.0)	6 (17.1)
AST (U/L)	39.2 (14.0–224.0)	33.8 (14.0–58.0)	47.1 (19.0–224.0)	7 (20.0)
ALT (U/L)	46.2 (8.0–372.0)	31.8 (12.0–62.0)	58.3 (8.0–372.0)	7 (20.0)
Albumin (g/L)	42.1 (32.5–47.6)	40.0 (35.5–44.5)	42.6 (32.5–47.6)	18 (60.0)
Morbidity, n (%)				
No morbidity	14 (40.0)	3 (30.0)	11 (44.0)	
Grade I	7 (20.0)	1 (10.0)	6 (24.0)	
Grade II	8 (22.9)	4 (40.0)	4 (16.0)	
Grade III	5 (14.2)	1 (10.0)	4 (16.0)	
Grade IV	0 (0.0)	0 (0.0)	0 (0.0)	
Grade V	1 (2.9)	1 (10.0)	0 (0.0)	
Postoperative stay				
ICU (d)	1.5 (0.0–15.0)	2.8 (0.0–15.0)	1.0 (0.0–8.0)	
**Total hospitalization (d)**	13.5 (4.0–75.0)	14.5 (5.0–34.0)	13.3 (4.0–75.0)	
PHLF ISGLS, n (%)				
**No PHLF**	25 (71.4)	0 (0.0)	25 (100.0)	
**Grade A**	5 (14.3)	5 (50.0)		
**Grade B**	4 (11.4)	4 (40.0)		
**Grade C**	1 (2.9)	1 (10.0)		

*Note*: Values in bold: PHLF vs. noPHLF: *p*>0.05.

ALT, alanine aminotransferase; AP, alkaline phosphatase; AST, aspartate aminotransferase; CCC, cholangiocellular carcinoma; CASH, chemotherapy-induced acute steatohepatitis; CTx, chemotherapy; GGT, gamma-glutamyl transpeptidase; ICU, intensive care unit; ISGLS, International Study Group of Liver Surgery; mCRC, metastatic colorectal cancer; PDR, plasma disappearance rate; PHLF, posthepatectomy liver failure; PT, prothrombin time; RBCs, red blood cells; SB, serum bilirubin.

### Neutrophil accumulation and NET formation in the regenerating liver is elevated in patients who develop PHLF

**FIGURE 1:**
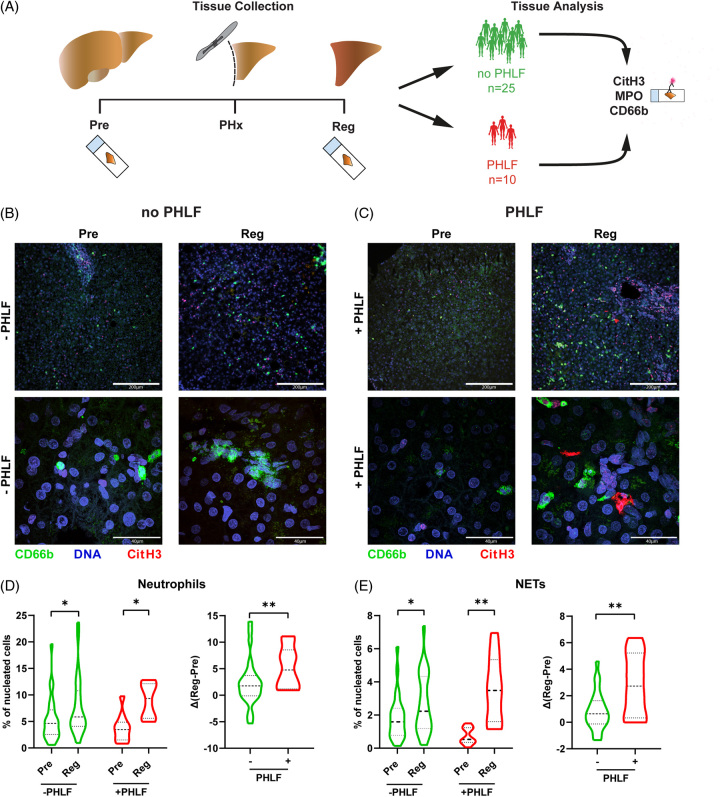
Neutrophils and NETs in PHLF. (A) Experimental scheme. Liver biopsies were collected before (Pre) and 2 hours after (Reg) PHx from 25 patients without PHLF and 10 patients with PHLF. (B, C) Immunofluorescence staining of DNA (Hoechst33342), neutrophils (CD66b), and NETs (CitH3) of 35 hepatic resection biopsies taken before resection (Pre) and 2 hours after resection (Reg). Pictures are representative of (B) 25 patients without PHLF or (C) 10 patients with PHLF. (D, E) Relative quantification of (D) neutrophils and (E) NETs in patients with PHLF or without PHLF (two-way ANOVA with Tukey correction for multiple comparisons: *p<0.05, **p<0.01). Regeneration-induced effects depicted as difference after-before resection (∆Reg-Pre) (unpaired *t* test: **p <0.01). Abbreviations: CitH3, citrullinated histone H3; MPO, myeloperoxidase; NET, neutrophil extracellular trap; PHLF, posthepatectomy liver failure; PHx, partial hepatectomy.

Analysis of liver biopsies before (Pre) and 2 hours after ligation (Reg) (Figure 1A) revealed that neutrophil infiltration (MPO^+^ CD66b^+^) as well as NET formation (MPO^+^ CD66b^+^ CitH3^+^) was significantly increased in the regenerating liver tissue samples (Supplemental Figure S1, http://links.lww.com/HC9/A700).

Next, we investigated the association of neutrophil infiltration and NET formation on patient outcomes. While patients with and without PHLF showed an increase in neutrophils and NETs 2 hours after hepatic resection, we observed more influx of total and CitH3^+^ neutrophils in liver tissues from patients with PHLF than patients without PHLF, indicating higher NET formation (Figures 1B, C). Quantification revealed a significant increase of neutrophil accumulation 2 hours after induction of liver regeneration in both groups (no PHLF: *p*=0.024; PHLF: *p*=0.036) (Figure 1D, left). Moreover, comparing regeneration-induced differences (Δ(Reg-Pre)) of PHLF versus no PHLF biopsies revealed that patients with PHLF showed a higher influx of neutrophils after liver resection compared to patients with normal functional liver regeneration (mean no-PHLF vs. PHLF: 2.333 vs. 5.277; *p*=0.0488) (Figure 1D, right). Similar results were observed with regard to NET formation during early liver regeneration. Importantly, while both groups showed a significant increase, we observed significantly more NET-forming neutrophils in patients with PHLF (no PHLF Pre vs. Reg: *p*=0.0242.; PHLF Pre vs. Reg: *p*=0.0018; Δ(Reg-Pre) no PHLF vs. PHLF: *p*=0.0043; Figure 1E). Interestingly, more CitH3 signal was observed around the vessels, indicating that especially infiltrating neutrophils form NETs. Of note, patients with different tumor entities, ages, genders, and specific ISGLS scores did not show any significant differences in markers of neutrophil infiltration or NET formation in tissue or plasma (Supplemental Figure S2, http://links.lww.com/HC9/A700).

### Extracellular traps in the liver predominantly derive from neutrophils and not macrophages, mast cells, or eosinophils

**FIGURE 2:**
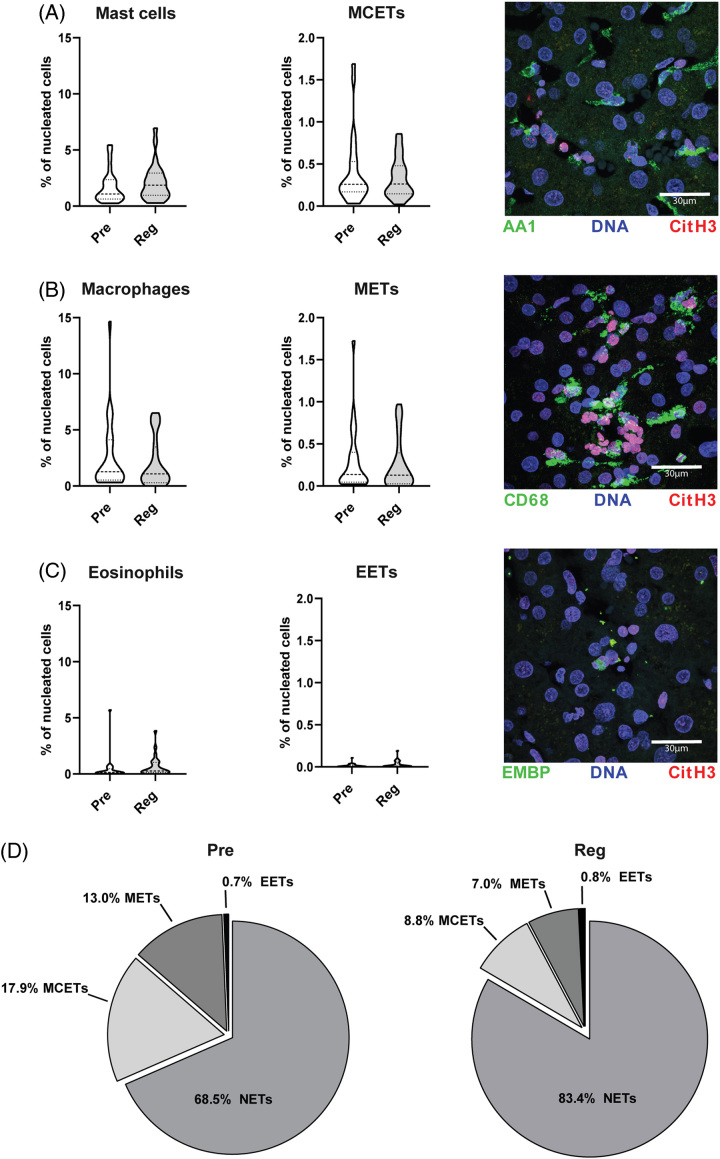
Extracellular traps derived from mast cells (MCETs), macrophages (METs), and eosinophils (EETs) in liver regeneration. Relative quantification of immunofluorescence staining of DNA (Hoechst33342). (A) Mast cells (AA1), (B) macrophages (CD68), or (C) eosinophils (EMBP) and their respective extracellular traps via CitH3 of 25 hepatic resection biopsies taken before (Pre) or after resection (Reg). Representative pictures given. (D) Comparison of NETs, MCETs, METs, and EETs Pre vs. Reg in percentage of all extracellular traps (unpaired *t* test). Abbreviations: CitH3, citrullinated histone H3; EET, eosinophil extracellular trap; MCET, mast cell extracellular trap; MET, macrophage extracellular trap; NET, neutrophil extracellular trap.

As extracellular traps can also derive from other cell types than neutrophils, we also analyzed extracellular trap formation by mast cells (mast cell extracellular traps), macrophages (macrophage extracellular traps), and eosinophils(eosinophil extracellular traps) during liver regeneration in a subset of 25 patients. Although we detected a small amount of extracellular traps from these cell types, the numbers of macrophages, mast cells, and eosinophils were not significantly altered 2 hours after ligation of the portal vein relative to before ligation. Furthermore, no significant differences could be observed in their respective ETs Pre vs. Reg (Figures 2A–C). Accordingly, ETs in the liver tissue are predominantly derived from neutrophils (Figure 2D). ETs derived from macrophages, mast cells, and eosinophils were only present to a minor extent in the liver tissue and made up an even smaller portion postoperatively. Lastly, induction of cell influx and ET formation of macrophages, mast cells, and eosinophils were not significantly altered between patients undergoing PHLF and patients with functional liver regeneration (Supplemental Figure S3, http://links.lww.com/HC9/A700).

### High levels of plasma MPO are associated with liver failure after hepatic resection and identify patients at high risk for PHLF

**FIGURE 3:**
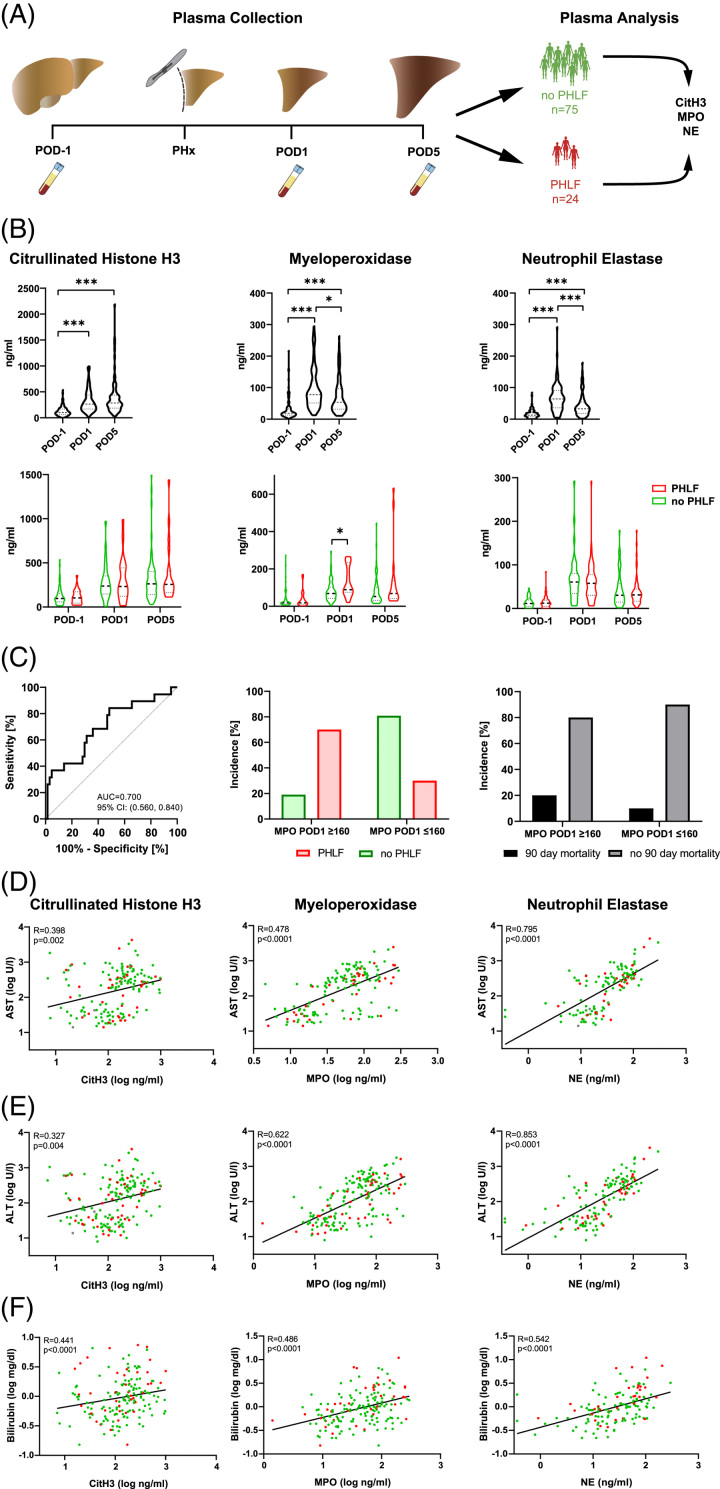
Plasma levels of CitH3, MPO, and NE are regulated in liver regeneration. (A) Experimental scheme. Plasma was collected 1 day before (POD−1), 1 (POD1) and 5 days after (POD5) PHx from 75 patients without PHLF and 24 patients with PHLF. (B) Upper panel: CitH3, MPO, and NE levels on POD−1, POD1 and POD5 (one-way ANOVA: *p<0.05, ***p<0.001). Lower panel: CitH3, MPO, and NE levels in patients with vs. without PHLF (two-way ANOVA: *p<0.05). (C) ROC curve comparing the predictive potential of MPO POD1 with the prevalence of PHLF (left panel); incidence [%] of PHLF (middle panel) and 90-day mortality (right panel) in high-risk patient groups (defined by a cutoff of MPO POD1 levels of above 160 ng/mL). (D–F) Correlation of plasma CitH3, MPO, and NE POD1 with AST, ALT, and bilirubin POD1 plasma levels. Abbreviations: ALT, alanine-aminotransferase; AST, aspartate aminotransferase; CitH3, citrullinated histone H3; MPO, myeloperoxidase; NE, neutrophil elastase; PHLF, post hepatectomy liver failure; PHx, partial hepatectomy; ROC, receiver operating characteristic.

Next, we assessed if surrogate markers for NETs and neutrophil activation were also detectable in patient’s blood. Therefore, we analyzed plasma samples from 99 patients (of which 24 patients experienced PHLF) undergoing hepatic resection taken on the day before surgery (POD−1) as well as 1 and 5 days after operation (POD1 and POD5, respectively), for neutrophil activation and NET formation (CitH3, MPO, and NE) (Figure 3A). All three plasma markers showed significant increases on POD1 and POD5 (CitH3: *p*<0.001, Figure 3B). Using this cutoff, a high-risk patient group could be identified, with 70% of patients above the cutoff developing PHLF. PHLF is associated with increased risk for short-term postoperative mortality^18^ and indeed, 91.6% of patients below our high-risk cutoff did not develop 90-day postoperative mortality (Figure 3C).

### Plasma markers for neutrophil activation and NET formation correlate with liver injury

Further, we observed strong correlations between plasma levels of CitH3, MPO, and NE with classical markers of liver tissue injury such as aspartate aminotransferase and ALT (Figures 3D, E). Similarly, bilirubin plasma levels significantly correlated with plasma levels of CitH3, MPO, or NE (Figure 3F). In contrast, overall platelet counts or prothrombin time did not correlate with markers of NET formation (Supplemental Figure S3, http://links.lww.com/HC9/A700, Supplemental Figure S4, http://links.lww.com/HC9/A700).

### Inhibition of NETs improves levels of cell cycle markers KI67, Cyclin D1, and PCNA in an FFD PHx mouse model

**FIGURE 4:**
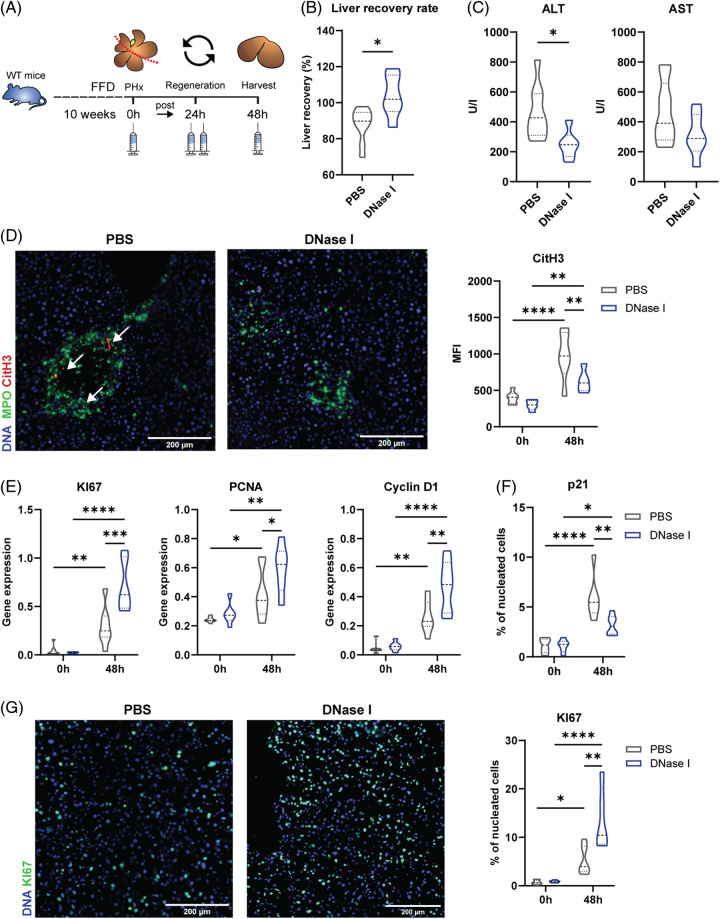
Reduction of NETs through DNase I in an FFD PHx mouse model. (A) Experimental scheme. Fifteen-week-old WT mice on FFD were treated with PBS or DNase I before being subjected to 70% PHx. (B) Remnant liver recovery rate. (C) AST and ALT measurements after PHx. (D) Immunofluorescence staining and relative quantifications of CitH3 and MPO in mouse hepatic sections before and after PHx. (E) Gene expression levels of KI67, PCNA, and Cyclin D1 in harvested mouse liver tissues before and 48 hours after PHx. (F, G) Immunofluorescence staining and relative quantifications of (F) p21 and (G) KI67 in mouse hepatic sections before and after PHx. (one-way ANOVA: *p <0.05, **p <0.01; n=6–7). Abbreviations: ALT, alanine-aminotransferase; AST, aspartate aminotransferase; CitH3, citrullinated histone H3; FFD, fast-food diet; MPO, myeloperoxidase; NET, neutrophil extracellular trap; PHx, partial hepatectomy; WT, wild type.

To unravel a causal relationship between NETs and host damage and to evaluate the therapeutic potential of NET inhibitors in liver regeneration, we performed a mouse model of metabolically challenged mice that were treated with DNase I or PBS before undergoing PHx (Figure 4A). When comparing the liver recovery rate of mice with and without NET inhibition, we found potential beneficial effects of NET inhibition on liver regeneration (PBS vs. DNase I: *p*=0.0127) (Figure 4B). This was further confirmed by the decrease in ALT, indicating less liver damage (ALT: PBS vs. DNase I: 0.0208; Figure 4C). Reduction of NETs by Dnase I was confirmed by significantly diminished immunofluorescence signals for MPO and CitH3 48 hours after PHx (PBS vs. Dnase I: 84.2%) (Figure 4D, MPO: quantification data not shown). Improved proliferation could be identified by increased gene expression levels of cell cycle markers KI67, PCNA, and Cyclin D1 at 48 hours after surgery (KI67: PBS vs. Dnase I: 0.0002; PCNA: PBS vs. Dnase I: 0.0325; Cyclin D1: PBS vs. Dnase I: 0.0014) (Figure 4E). Cell proliferation was further confirmed on a protein level by immunofluorescence staining for KI67 and the cell cycle arrest marker p21 (Figures 4F, G), which revealed a 2.6-fold increase in KI67 levels and a 0.53-fold decrease in p21 levels 48 hours after PHx of mice receiving Dnase I (KI67: *p*=0.0012; p21: *p*=0.0017).

## DISCUSSION

While rodent models of liver regeneration taught us that neutrophils are indispensable during the priming phase of liver regeneration, we are able to document that excessive intrahepatic accumulation of neutrophils bears deleterious features and correlates with markers of intrahepatic cell death in humans. In patients undergoing PHx, we observed rapid and frequent intrahepatic NET formation, which was elevated in patients with dysfunctional liver regeneration, suggesting that NETs might have a critical negative effect on liver regeneration. Ultimately, we characterized a high-risk group of patients that could potentially benefit from an NET-targeted treatment approach. Moreover, in a murine PHx model, we could show that the inhibition of NET accelerated hepatocyte proliferation and liver regeneration.

Previous observations by Selzner et al^21^ Demonstrated delayed liver regeneration and reduced liver-specific TNF-α and IL-6 levels in a neutropenic mouse model and proposed that binding of neutrophils to hepatocytes through ICAM-1 after hepatectomy triggers hepatocyte proliferation by Kupffer cells. In line, we and others could also show the beneficial effects of neutrophils in patients and rats in liver regeneration. In the late stages of liver regeneration, neutrophils switch toward a proregenerative phenotype and they also protect individuals from infiltrating gut-derived endotoxins after hepatic resection.^22–24^ However, our present study clearly demonstrates that neutrophil infiltration has to be well-balanced as excessive infiltration of neutrophils and NET formation is associated with adverse outcome after hepatic resection. Overactivation of neutrophils drives sterile liver inflammation, hepatocyte death, and steatosis through the production of reactive oxygen species.^25^ NET formation meditates the death of hepatocytes in vitro^26^ and may cause nuclear DNA damage and the loss of mitochondrial integrity of hepatocytes^27^ as well as damage to endothelial cells and Kupffer cells.^26,28^ Furthermore, Von Meijenfeldt et al^16^ emphasize the aspects that the formation of NETs is tied to a lesional process mediated by neutrophil trapping in ALF as well as the adverse impact of NETs on clinical outcome. In our cohort, however, the phenomenon could be linked to a surgical hit, specifically, the reduction in liver volume following surgical resection. This reduction in volume could expose the residual parenchyma or functional liver remnant to an increased portal hyperflow, subsequently initiating regeneration and potentially playing a role in the mediation of a “Small for Size” phenomenon and thereby a possible explanation why excessive neutrophil and NET expression could also impair the regeneration process after PHx.

We could further show that in patients undergoing liver resection plasma levels of CitH3, MPO, and NE correlate with hepatic dysfunction markers including aspartate aminotransferase, ALT, and bilirubin, further confirming a possible functional role of neutrophils and NETs in liver diseases. Our data are in line with the hypothesis that not only reactive oxygen species but also neutrophil-derived proteases, such as MPO and NE, contribute to hepatocyte death^29,30^ as also neutrophil proteases mediate direct hepatotoxicity in co-culture experiments of hepatocytes with activated neutrophils.^31,32^ In particular, NE participates in the early steps of the inflammation cascade in viral acute hepatitis, and elastase inhibitors significantly reduced the expression of hepatic inflammatory mediators.^29^

In addition, we provide the first histological evidence for the presence of mast cell, macrophage, and eosinophil extracellular traps in liver tissue, indicating that not only neutrophils are capable of producing extracellular traps during liver regeneration. However, we detected no changes in these ETs within the first 2 hours after liver resection, suggesting a minor role of these ETs during the initiation phase of liver regeneration.

Almost all patients undergoing liver resection show signs of underlying liver damage. To mimic this in a rodent model, we choose a well-characterized metabolically induced liver inflammation model, with high calories, high cholesterol, and high fructose intake, which ultimately leads to NASH development.^33^ We performed PHx after 10 weeks of an FFD, where hepatic lipid composition changes and hepatic inflammation already occurs but NASH is not yet fully developed, which we regarded to most closely mimic underlying liver disease in the majority of our patients.

In this mouse model of metabolically induced liver inflammation, we could demonstrate that targeting of NETs directly by DNase I increased hepatic cell proliferation and liver regeneration, indicating that NETs actively impede the regenerative potential in the remnant liver. This is in line with several animal experiments examining the harmful involvement of NETs in other liver diseases that involve repair processes, including acute liver failure, viral hepatitis, alcohol-associated hepatitis, liver cancer, hepatic ischemia/reperfusion injury, and liver transplantation.^11,34–41^

NET degradation represents a safe treatment option as drugs like DNase I are already used in other diseases such as cystic fibrosis and off-label use for COVID-19.^42,43^ Furthermore, heparin or colchicine administration presents options to interfere with NET formation by inhibiting histone-induced coagulation or actin cytoskeleton remodeling in NET-forming neutrophils, respectively.^44,45^ Lastly, novel inhibitors interfering with MPO or PAD4 represent potential therapeutic drugs. Indeed, the efficacy of these inhibitors was confirmed in various inflammatory disease models, such as hepatic ischemia/reperfusion injury, vasculitis, and systemic lupus erythematosus, proposing their potential beneficial effect on liver regeneration.^26,46,47^ Accumulating evidence indicates the beneficial influence of anti-inflammatory drugs on the postsurgical regeneration process.^48^ In a recent study, the administration of preoperative single-dose methylprednisolone to patients undergoing major liver resection led to lower postoperative complications and organ infections.^49^ Even specific NET-targeted therapies, using an NE inhibitor, have shown some benefit in patients undergoing hepatic resection; however, the overall extent of treatment effect was relatively minimal.^50^ This lack of treatment effect might have one critical reason. Indeed, PHLF occurs in about 5%–15% of patients after hepatectomy. Therefore, a universal treatment of all patients might mask the potential benefits of a certain treatment, given that the majority of patients will regenerate without any issues. Accordingly, we tried to assess if a neutrophil activation marker like MPO was able to identify patients who will develop PHLF and might therefore benefit from an NET-targeted therapy. Indeed, the risk of PHLF increased to about 70% in patients exceeding our MPO cutoff, indicating that these patients might benefit from a treatment supporting hepatic regeneration. More importantly, as MPO reflects a critical process dysregulated during human hepatic regeneration, as we show in these analyses, these patients might particularly benefit from an NET-targeted treatment.

Taken together, our human data and the results of rodent experiments emphasize the importance of a well-balanced neutrophil response for successful hepatic regeneration as excessive intrahepatic neutrophil accumulation and NET formation are associated with PHLF and circulating markers of neutrophil activation correlate with markers of intrahepatic injury. Importantly, MPO, as a circulating marker of NET formation, could help to identify patients at the highest risk of PHLF to allow for personalized hepatic regeneration treatments.

## Supplementary Material

SUPPLEMENTARY MATERIAL
