# Supplementary material for: Intrahepatic neutrophil accumulation and extracellular trap formation are associated with posthepatectomy liver failure
Source: Hepatol Commun. 2023 Dec 15;8(1):e0348. doi: 10.1097/HC9.0000000000000348 (PMC10727591; doi:10.1097/HC9.0000000000000348)
Supplement: SUPPLEMENTARY MATERIAL [file hc9-8-e0348-s001.docx]

**­­­­Supplementary data:**

Intrahepatic neutrophil accumulation and neutrophil extracellular trap formation is associated with post hepatectomy liver failure

Laura Brunnthaler^1^, Jonas Santol^1,7^, David Pereyra^2^, Miriam Brenner^1^, Lukas Herrmann^1^, Waltraud C. Schrottmaier^1^, Anita Pirabe^1^, Anna Schmuckenschlager^1^, Sarang Kim^2^, Anna Emilia Kern^2^, Felix Xaver Huber^2^, Lisa Emilie Michels^1^, Christine Brostjan^3^, Manuel Salzmann^4^, Philipp Hohensinner^5^, Renate Kain^6^, Thomas Gruenberger^7^, Patrick Starlinger^8*^, Alice Assinger^1*^

^1^Department of Vascular Biology and Thrombosis Research, Centre of Physiology and Pharmacology, Medical University of Vienna, Vienna, Austria

^2^Department of General Surgery, Division of Visceral Surgery, Medical University of Vienna, General Hospital, Vienna, Austria

^3^Department of General Surgery, Division of Vascular Surgery, Medical University of Vienna, General Hospital, Vienna, Austria

^4^Department of Medicine II, Division of Cardiology, Medical University of Vienna, General Hospital, Vienna, Austria

^5^Center for Biomedical Research, Medical University of Vienna, Austria

^6^Department of Pathology, Medical University of Vienna, General Hospital, Vienna, Austria

^7^Department of Surgery, HPB Center, Viennese Health Network, Clinic Favoriten and Sigmund Freud Private University, Vienna, Austria

^8^Department of Surgery, Division of Hepatobiliary and Pancreatic Surgery, Mayo Clinic, Rochester, MN, USA

*shared correspondence

**Supplementary Table 1: Primary and secondary antibodies used for immunofluorescence analyzes**

| Primary antibodies | Clone | Manufacturer | Cat # | Dilution factor |
| --- | --- | --- | --- | --- |
| Mouse anti-CD66b | 80H3 | Invitrogen | MA1-26144 | 1:200 |
| Mouse anti-CD68 | KP1 | Santa Cruz Biotechnology | Sc-200600 | 1:100 |
| Rabbit anti-Citrullinated Histone H3 (CitH3) | Polyclonal | Abcam | Ab51031 | 1:500 |
| Mouse anti-Eosinophil major basic protein (EMBP) | BMK-13 | Biorad | MCA5751 | 1:50 |
| Mouse anti-Mast Cell Tryptase (MCT) | AA1 | Bio-Rad Antibodies | MCA1438 | 1:250 |
| Goat anti-Myeloperoxidase (MPO) | Polyclonal | R&D systems | AF3667-SP | 1:80 |
| Rat anti-KI-67 | SolA15 | Invitrogen | 14-5698-82 | 1:100 |
| Rabbit anti-p21 | 2H2L13 | Invitrogen | 701151 | 1:200 |
|  |  |  |  |  |
| Secondary antibodies |  | Manufacturer | Cat # | Dilution factor |
| Chicken anti-Goat AF488 |  | Invitrogen | A21467 | 1:100 |
| Donkey anti-Mouse AF555 |  | Invitrogen | A31570 | 1:200 |
| Donkey anti-Rabbit Dylight650 |  | Abcam | Ab96922 | 1:100 |
| Donkey anti-Goat Dylight550 |  | Abcam | ab96936 | 1:100 |
| Donkey anti-Rat Dylight550 |  | Invitrogen | SA510027 | 1:100 |

**Supplementary Table 2: Designed primer used for real-time qPCR analysis**

| Primer | Fwd | Rev |
| --- | --- | --- |
| HPRT | 5′-TCAGTCAACGGGGGACATAAA-3′ | 5′-GGGGCTGTACTGCTTAACCAG-3′ |
| KI-67 | 5′-AATCCAACTCAAGTAAACGGGG-3′ | 5′-TTGGCTTGCTTCCATCCTCA-3′ |
| Proliferating cell nuclear antigen (PCNA) | 5′-GAACCTCACCAGCATGTCCA-3′ | rev 5′-ATTCACCCGACGGCATCTTT-3′ |
| Cyclin D1 | 5′- CTGGATGCTGGAGGTCTGTG-3′ | 5′- TCATCCGCCTCTGGCATTTT-3′ |

**Material & Methods**

*Scanning electron microscopy*

Tissue samples were microperfused with 2.5% glutaraldehyde in phosphate buffered saline (PBS) using a 27-gauge needle. Subsequently, liver tissue was sliced using fresh scalpels and continued fixation in 2.5% glutaraldehyde at 4°C was performed until further processing. After washing with PBS, tissue was dehydrated using a graded ethanol series (30%, 50%, 70%, 90%, 3x96%) followed by two desiccation steps in hexamethyldisilazane (HMDS). After dehydration, conductive silver was applied and a gold sputter deposition layer was coated onto the slide Finally, tissues were analyzed by scanning electron microscopy (JEOL LSM 5400).


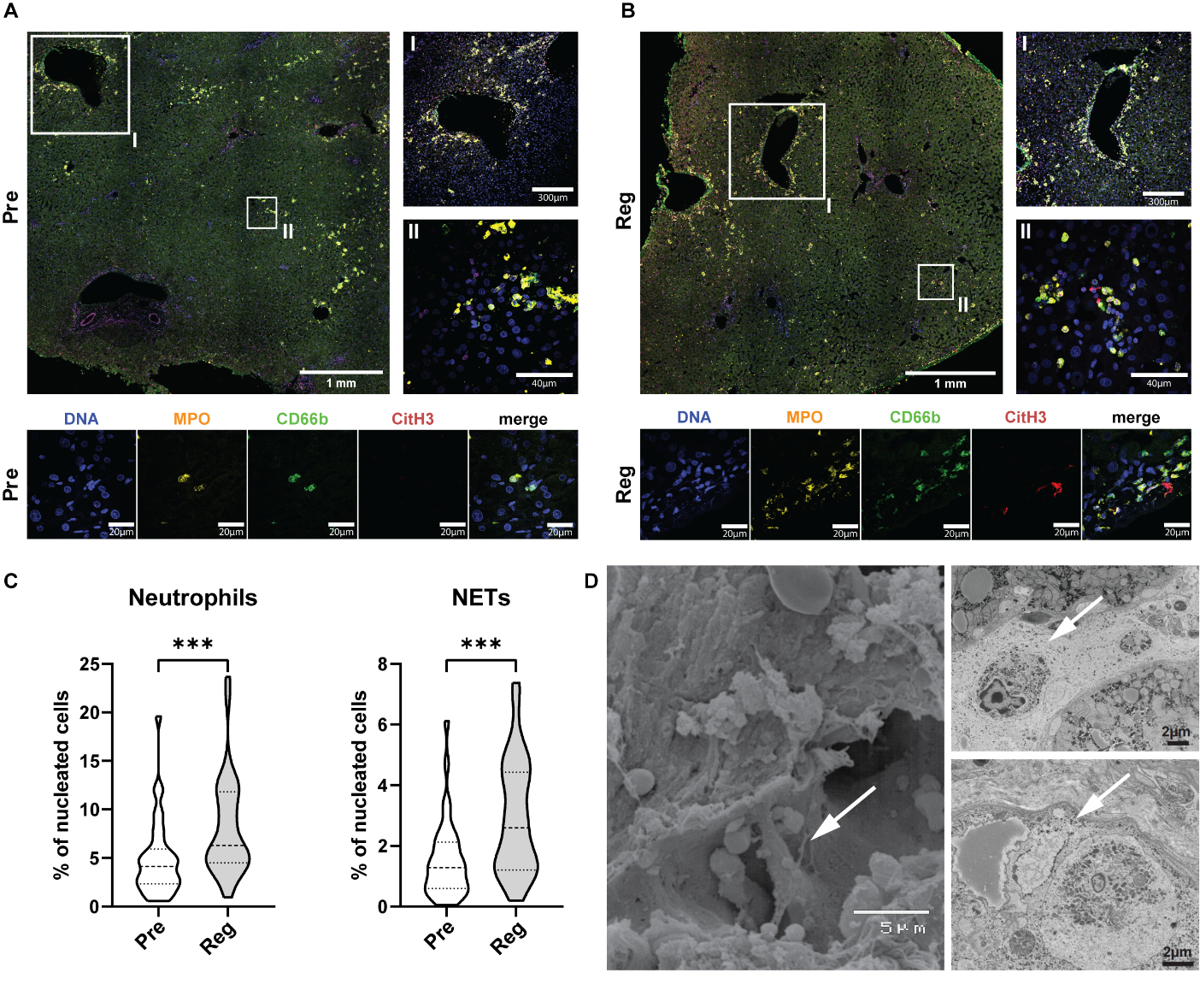


**Suppl. Fig. 1: Immunofluorescence analysis of neutrophils and neutrophil extracellular traps (NETs) in liver regeneration.** (A+B) Liver biopsies were collected before (Pre) and 2h post (Reg) partial hepatectomy (PHx) from 25 patients without post-hepatectomy liver failure (PHLF) and 10 patients with PHLF. Immunofluorescence staining of liver biopsies (A) Pre and (B) Reg showing DNA (Hoechst33342), myeloperoxidase (MPO), neutrophils (CD66b) and NETs (citrullinated histone H3; CitH3). (C) Relative quantification of neutrophils and NETs (Paired parametric t-test: ***p<0.001). (D) Electron microscopy images of NET forming neutrophils in a regenerating liver sample.

***
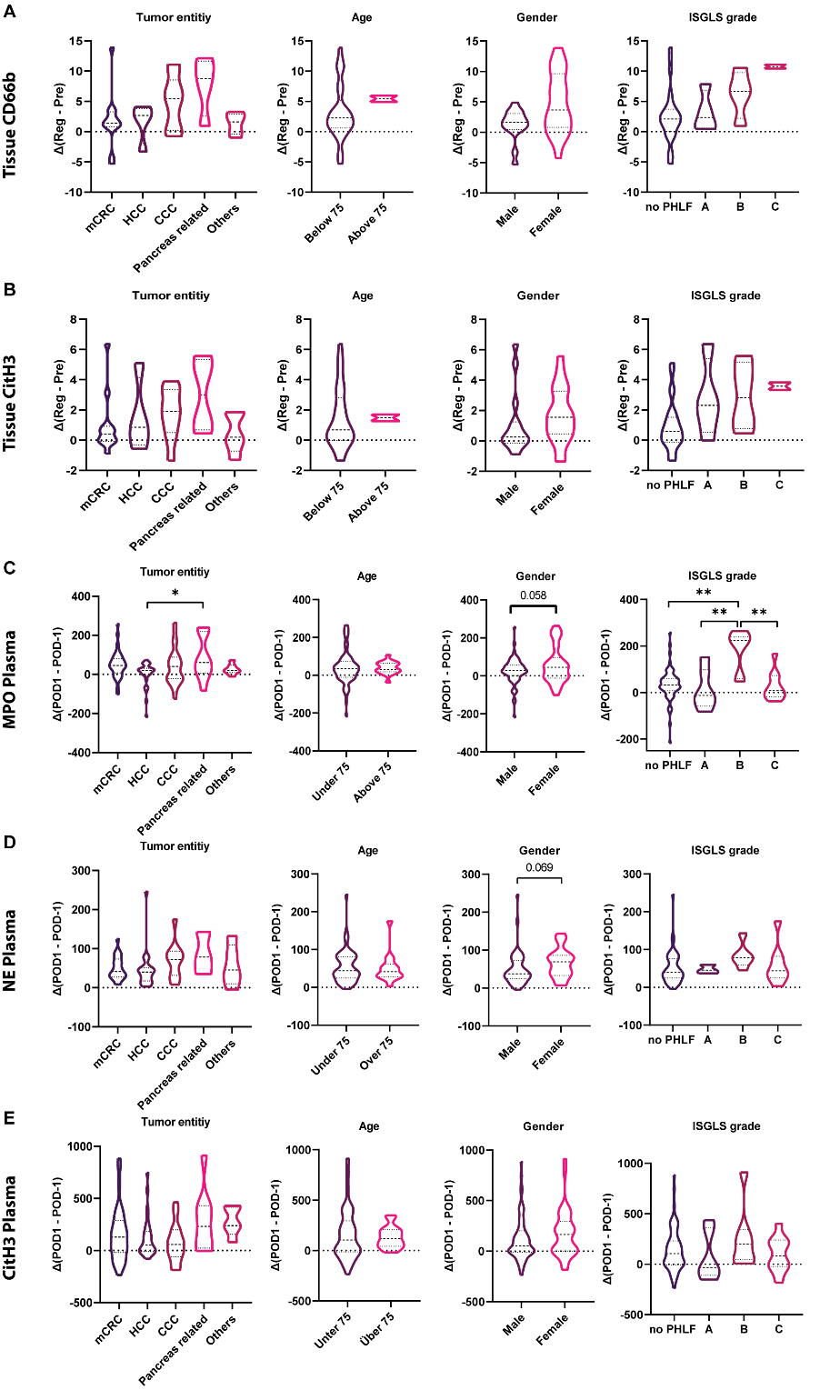
***

**Suppl. Fig. 2: Sub-analysis of neutrophil and neutrophil extracellular trap (NET) markers in patients according to tumor entity, age, gender and specific ISGLS score.** Sub-analysis of (A) neutrophils (CD66b) and (B) NETs (citrullinated histone H3; CitH3) assessed in liver biopsies collected before (Pre) and 2h post (Reg) partial hepatectomy (PHx) (n=25). Sub-analysis of (C) myeloperoxidase (MPO), (D) neutrophil elastase (NE) and (E) CitH3 levels in plasma collected one day before (POD-1) and one day (POD1) after the PHx from 99 patients. Differences of POD1 – POD-1 depicted as delta (∆) (tumor entity and ISGLS score: Two-way ANOVA: *p<0.05, **p<0.01; age and gender: unpaired parametric t-test).

**
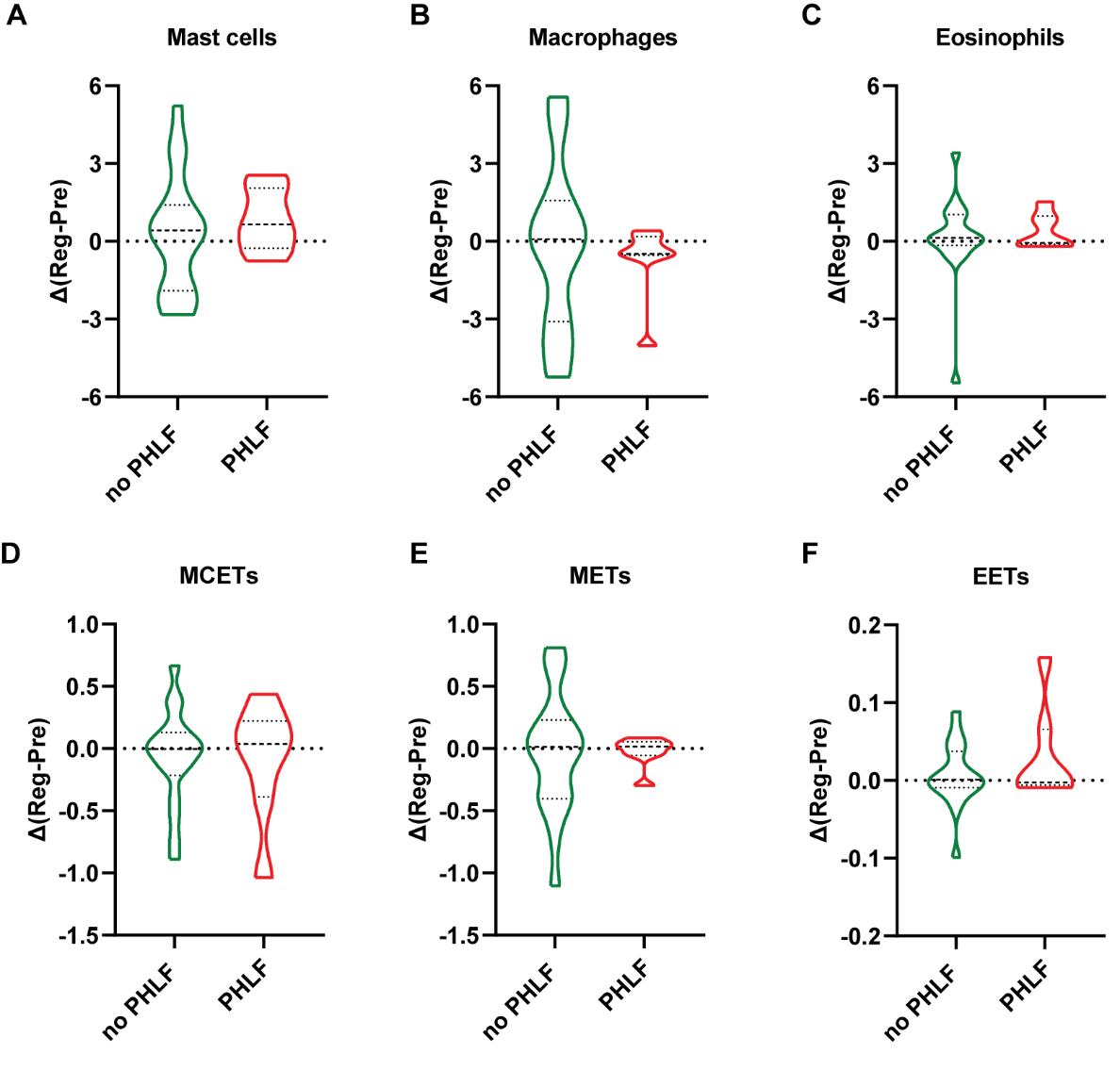
**

**Suppl. Fig. 3: Extracellular traps derived from mast cells (MCETs), macrophages (METs) and eosinophils (EETs) in post-hepatectomy liver failure (PHLF).** Relative quantification of (A) mast cells, (B) macrophages or (C) eosinophils and their respective extracellular traps (D-F) in liver biopsies of patients with PHLF vs. no PHLF (Unpaired t-test). Tissue samples were collected before (Pre) and 2h post (Reg) partial hepatectomy (PHx). N=18 patients without PHLF and 7 patients with PHLF.

**
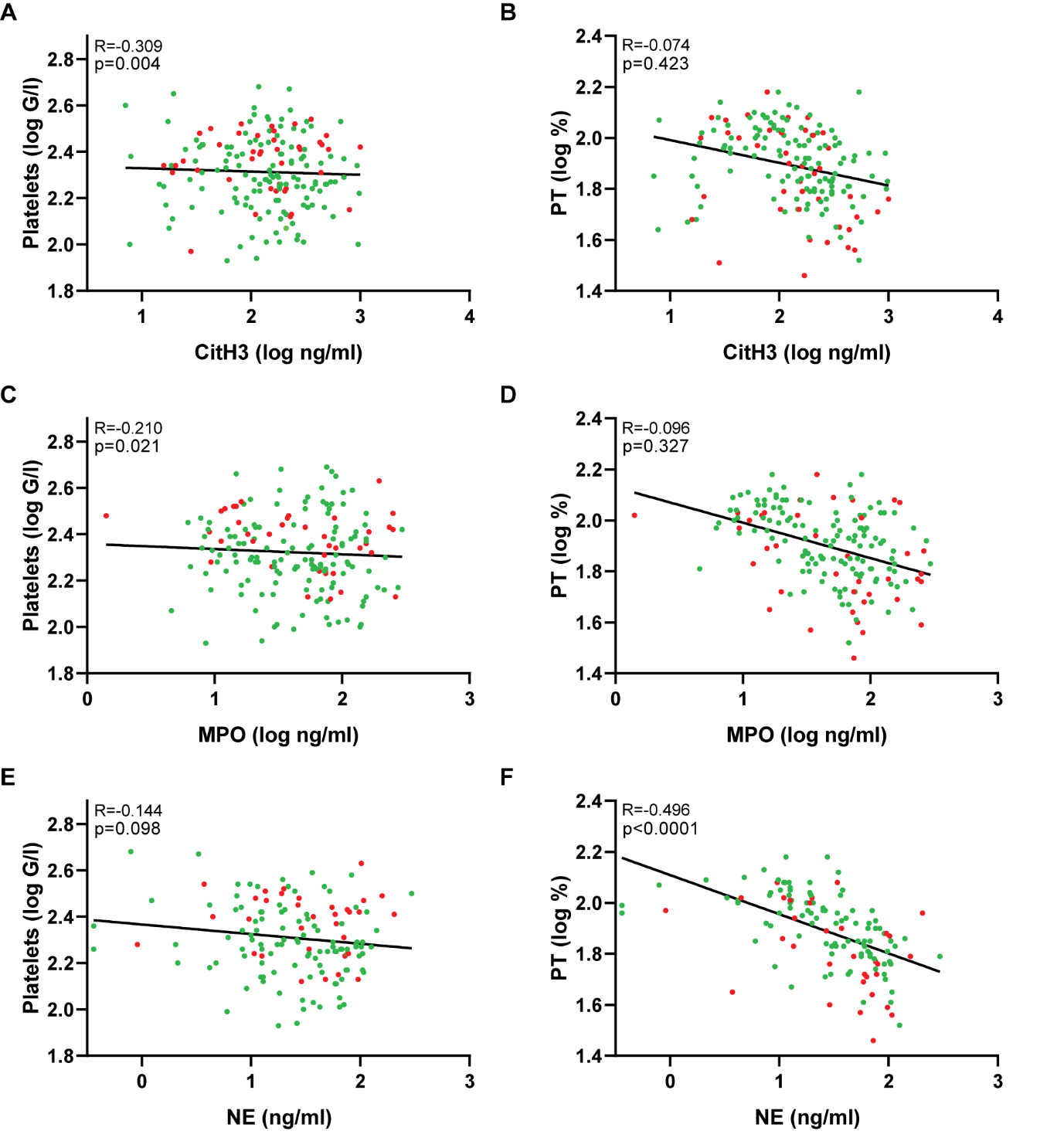
**

**Suppl. Fig. 4: Citrullinated histone H3 (CitH3), myeloperoxidase (MPO) and neutrophil elastase (NE) plasma levels do not correlate with platelet numbers and prothrombin time (PT) in liver regeneration.** Correlation of (A+B) CitH3, (C+D) MPO and (E+F) NE plasma levels with (A, C, E) platelet counts and (B, D, F) PT levels (Data was log transformed). Plasma was collected one day before (POD-1), one (POD1) and five days after (POD5) partial hepatectomy in 75 patients without post hepatectomy liver failure (PHLF) (green) and 24 patients with PHLF (red).
